# Reduced Thalamic and Pontine Connectivity in Kleine–Levin Syndrome

**DOI:** 10.3389/fneur.2014.00042

**Published:** 2014-04-02

**Authors:** Maria Engström, Thomas Karlsson, Anne-Marie Landtblom

**Affiliations:** ^1^Division of Radiological Sciences, Department of Medical and Health Sciences (IMH), Linköping University, Linköping, Sweden; ^2^Center for Medical Image Science and Visualization (CMIV), Linköping University, Linköping, Sweden; ^3^Division of Disability Research and Linnaeus Centre HEAD, Department of Behavioral Science and Learning, Linköping University, Linköping, Sweden; ^4^Division of Neurology, Department of Clinical and Experimental Medicine (IKE), Linköping University, Linköping, Sweden

**Keywords:** periodic idiopathic hypersomnia, nystagmus, sleep, functional magnetic resonance imaging, brain stem, pons, thalamus, cerebellum

## Abstract

The Kleine–Levin syndrome (KLS) is a rare sleep disorder, characterized by exceptionally long sleep episodes. The neuropathology of the syndrome is unknown and treatment is often inadequate. The aim of the study was to improve understanding of the underlying neuropathology, related to cerebral networks, in KLS during sleep episodes. One patient with KLS and congenital nystagmus was investigated by resting state functional magnetic resonance imaging during both asymptomatic and hypersomnic periods. Fourteen healthy subjects were also investigated as control samples. Functional connectivity was assessed from seed regions of interest in the thalamus and the dorsal pons. Thalamic connectivity was normal in the asymptomatic patient whereas the connectivity between the brain stem, including dorsal pons, and the thalamus was diminished during hypersomnia. These results suggest that the patient’s nystagmus and hypersomnia might have their pathological origin in adjacent dorsal pontine regions. This finding provides additional knowledge of the cerebral networks involved in the neuropathology of this disabling disorder. Furthermore, these findings regarding a rare syndrome have broad implications, and results could be of interest to researchers and clinicians in the whole field of sleep medicine.

## Introduction

In the current study, we investigated the neuropathology of periodic idiopathic hypersomnia, or the Kleine–Levin syndrome (KLS), by imaging the functional connectivity of the thalamus in one KLS patient for whom results were possible to obtain during both hypersomnic and asymptomatic periods. We hypothesized that such an investigation could highlight the neural networks involved in the appearance of symptoms in KLS.

One female KLS patient was investigated by resting state functional magnetic resonance imaging (fMRI) during both hypersomnic and asymptomatic periods. The hypersomnic periods usually occur twice a year with characteristic duration of about 3 weeks. This patient has a severe character of her hypersomnic periods, initially with seemingly comatose episodes when she sometimes has to be enterally nourished. Several electroencephalogram (EEG) examinations, however, demonstrated awake activity. The initial evaluation suggested a psychogenic condition, but later the pattern of the KLS emerged. During the hypersomnic periods, the patient also has severe hyperacusis, which is not present during asymptomatic periods. As often noted in KLS, the patient has psychological symptoms during hypersomnia. The psychological symptoms in this patient are manifested by derealization and depersonalization, as well as a tendency to regressive behavior. She also has a certain sugar craving despite enteral feeding. The patient suffers from congenital spontaneous nystagmus, which is predominantly horizontal. The nystagmus is worsened during hypersomnia.

Structural neuroimaging, as assessed by computed tomography (CT) and MRI, is normal. On one occasion during an early symptomatic period, EEG from especially the bilateral temporal lobes showed low amplitudes. This low-amplitude EEG was expected because of the patient’s somnolence. However, also a focal theta–delta abnormality was observed in the left frontal lobe. At the very same locus, there was also a suspect epileptiform activity, but a diagnosis of epilepsy could be excluded. The neurophysiologist suggested a deep subfrontal left-sided lesion or disturbance, possibly including the amygdala, which could affect the patient’s emotional functions. Later, an investigation of the cerebral blood flow by single photon emission computed tomography (SPECT) revealed inter-episodic hypoperfusion in the left temporal lobe, as seen in many of our patients with KLS (1).

Neuropsychological testing has shown a cognitive profile within or just above normal performance, with the exception of reduced visual working memory capacity. The finding of inter-periodic dysfunctions of working memory is common in our patient material, but the character of the dysfunction can vary (1, 2). Neither clinical assessment nor the hospital anxiety and depression (HAD) scale indicated depression, anxiety disorder, or any other psychiatric diagnosis.

## Background

Kleine–Levin syndrome is a sleep disorder characterized by exceptionally long sleep episodes, which can endure up to several weeks and recur several times a year. During the hypersomnic periods, the KLS patients often suffer from behavioral, perceptual, and cognitive disturbances, such as binge eating, delusions, and memory problems (3, 4). Structural neuroimaging and inter-episodic EEG are usually normal. However, functional neuroimaging shows frontotemporal and thalamic abnormality that indicate complex disruptions in thalamocortical networks in episodes, but also partially between episodes (5–7). Functional MRI has shown hyperactivation in the left thalamus and abnormal coupling between the thalamus and the brain’s executive and salience networks in asymptomatic KLS patients during working memory performance (8–10). SPECT has shown hypoperfusion of the bilateral thalami during hypersomnic periods, which was not persistent during the asymptomatic periods (6, 11, 12). Despite convincing data indicating thalamic involvement, the neuropathology of KLS remains unknown.

## Subjects and Methods

### Subjects

One female KLS patient was included in the study. She was investigated by resting state fMRI at one occasion in asymptomatic state and at one occasion during hypersomnia. The patient was diagnosed with KLS at the age of 18 years, but with onset of symptoms at the age of 15 years. At the time of the investigation, she was 21 years. When the patient was investigated in the hypersomnic period, she was not able to perform the standard pre-fMRI cognitive tests or respond to the clinical interview. She had much problem with her nystagmus and wore eye shields. Nevertheless, she was able to walk into the scanner and she agreed to participate in the study.

In order to explore normal functional connectivity, we also investigated 14 healthy controls (7 females). The mean age of the controls was 24.6 years (SD = 6.7 years).

### Image acquisition and analysis

The KLS patient and the healthy controls were investigated with blood oxygen level dependent (BOLD) fMRI acquired on a Philips Achieva 1.5 T scanner, using following parameters: echo time, TE = 40 ms, repetition time, TR = 2700 ms, flip angle = 90°, number of slices = 32, voxel size = 3 mm × 3 mm × 3 mm, scanner mode = interleaved, number of dynamics = 80. The subjects were instructed to rest with their eyes closed during the examination.

Image preprocessing was performed using SPM8 software (Wellcome Department of Imaging Neuroscience, University College, London, UK). Images were realigned to correct for movement during scanning and normalized to Montreal Neurological Institute (MNI) template. The normalized images were smoothed with a full width half maximum (FWHM) Gaussian kernel of 8 mm.

Functional connectivity from a seed in the left thalamus was calculated using the RESTing state fMRI analysis toolkit (REST, resting-fmri.sourceforge.net). The linear trends were removed and the images were band pass filtered in the range 0.01–0.1 Hz. The seed region of interest (ROI) was constructed from a sphere with radius = 8 mm centered at the site of thalamic hyperactivation in KLS (8). Image voxels representing cerebrospinal fluid (CSF) were removed from the ROI. In a *post hoc* analysis, a second ROI was placed in the dorsal pons (radius = 8 mm), including image voxels that were connected to the thalamus in the healthy controls and the asymptomatic patient.

For each participant, the mean time courses within the thalamic and pontine ROIs were correlated to the average time courses in all image voxels of the whole brain. The individual correlation maps were normalized using Fisher’s *Z* transform. The *Z*-maps of the controls were entered a one-sample *t*-test to enable the visualization of regions with conjoint functional connectivity in healthy subjects.

The number of voxels that were functionally connected to the thalamic and pontine seeds was calculated using a ROI analysis. In the ROI analysis, we used predefined regions in the frontal, temporal, parietal, occipital, and limbic lobes and in the cerebellum, the brain stem, and the thalamus using the XjView tool (www.alivelearn.net/xjview).

## Results

**Figure 1:**
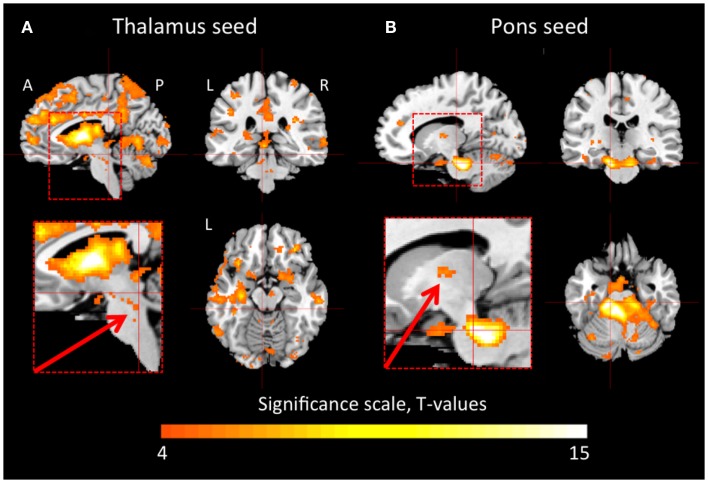
**Thalamic and pontine functional connectivity in healthy controls**. The images show regions that had significant inter-subject functional connectivity to the thalamus **(A)** and pons **(B)**, respectively. The color scale represents *t*-statistic values in the range of 4–15. A *t*-value of 4.22 corresponds to *p* < 0.001. The inserts show magnifications of the brain stem and thalamus. The arrows point to connections between the brain stem and the thalamus that were reduced in the patient during hypersomnia. A, anterior; P, posterior; L, left; R, right.

In the healthy controls, the thalamus was functionally connected to large portions of the cerebral cortex and the cerebellum (Figure 1A). There were also connections between areas in the brain stem, including the pons and the midbrain, and the thalamus. Figure 1B shows that the dorsal pons had strong connections to the cerebellum, but also to the thalamus. Furthermore, we observed functional connectivity between dorsal pons and the cortex, which is not clearly visualized in Figure 1B.

**Figure 2:**
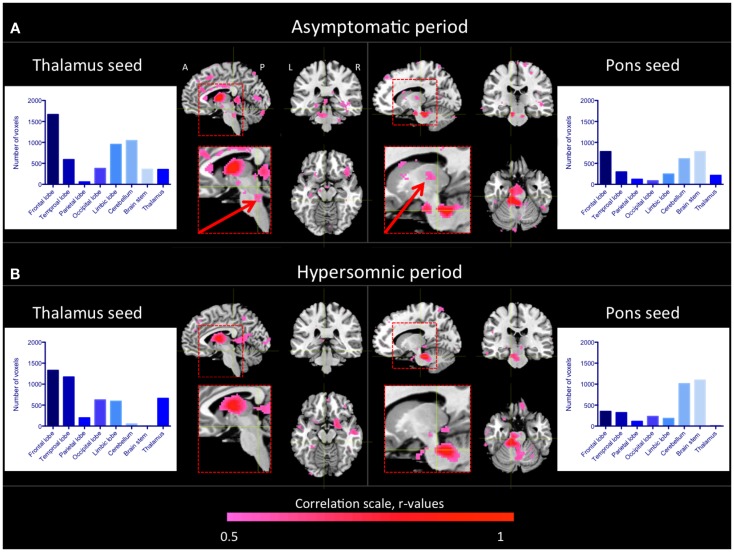
**Thalamic and pontine functional connectivity in the KLS patient during asymptomatic and hypersomnic periods**. The color scale represents correlation coefficients, *r* > 0.5. The inserts show magnifications of the brain stem and thalamus. The arrows point to connections between the brain stem and the thalamus that were reduced in the patient during hypersomnia. The histograms show the number of connected voxels to the thalamus and pons, respectively, during both asymptomatic **(A)** and hypersomnic **(B)** periods. A, anterior; P, posterior; L, left; R, right.

As seen in Figure 2A, similar regions as in healthy controls were functionally connected to the thalamus in the patient during the asymptomatic period. Especially, we observed functional connectivity between the brain stem, including the pons, and the thalamus as in the healthy subjects. When the patient was in a hypersomnic period, the connections between the thalamus and the cerebral cortex were largely intact, whereas the connections between the brain stem and the thalamus were substantially diminished (Figure 2B). The connections between the cerebellum and the thalamus were also diminished during hypersomnia.

When investigating the functional connectivity from a seed in the dorsal pons, we observed that the connections within the brain stem and to the cerebellum were basically preserved during hypersomnia.

## Discussion

The neuropathology of KLS is not known. Neuroimaging (6, 8, 10–12) and a few post mortem studies (13, 14) indicate possible thalamic pathology. In addition, patients with thalamic stroke show symptoms that resemble KLS (15). However, a recent study by us suggests that the hyperactivity in the thalamus could be a result of a secondary compensatory effect (9). In the current study, we investigated this issue further by studying thalamic and pontine functional connectivity in healthy subjects, and during asymptomatic and hypersomnic periods in one KLS patient. The results showed that the thalamus was, as expected, functionally connected to large portions of the cerebral cortex, the pons, and the midbrain in healthy subjects. Also in the asymptomatic patient, the thalamus was functionally connected to the cerebral cortex and the brain stem. On the other hand, during the hypersomnic period, the connections between the brain stem and the thalamus were substantially diminished. These results show that central areas of the ascending arousal system that regulates sleep and wakefulness were affected in the KLS patient during hypersomnia.

The ascending arousal system consists of a ventral pathway passing through the hypothalamus and a dorsal pathway passing through the thalamus (16, 17). A number of well-defined nuclei in the upper brain stem project to the hypothalamus via identified neurotransmitters, e.g., the locus coeruleus (noradrenaline), the raphe nuclei (serotonin), and the periaqueductal gray (dopamine). The input to the cortex from the ventral pathway is further facilitated by cholinergic, GABAergic, and glutamatergic neurons in the basal forebrain. The dorsal pathway connects to the thalamus from cholinergic neurons in the dorsal tegmentum and glutamatergic neurons in the dorsal pontine reticular formation, and also from brain stem monoaminergic neurons.

One limitation with the present study was that we were not able to control for the participants’ sleep and wakefulness during fMRI scanning. Sleep could be controlled by simultaneous EEG and fMRI measurements, a technique that was not implemented at the time of the study. Such measurements would allow for the comparison between functional connectivity during KLS hypersomnia and during normal sleep or hypersomnia induced by sleep deprivation. Thus, for the interpretation of our results, we cannot rule out that decreased functional connectivity between the brain stem and the thalamus in the hypersomnic KLS patient was not caused by her somnolence or sleep. In ongoing studies at our lab, we use EEG-fMRI to investigate differences between KLS hypersomnia, normal sleep, and fatigue.

Premotor neurons in the dorsal pontine reticular formation and medulla control horizontal eye movements (18–20). The patient in this study has congenital horizontal nystagmus, which indicates a possible influence from the medulla or the dorsal pontine reticular formation. Thus, the pontine reticular formation contains both neurons that regulate oculomotor function and neurons that regulate sleep and wakefulness. It is therefore tempting to suggest that the patient’s nystagmus and hypersomnia have their pathological origin in adjacent dorsal pontine regions.

We are aware of the limitations to make generalized conclusions from a single case study. Nevertheless, the findings in the present work provide as basis for future studies on the neuropathology in KLS.

## Concluding Remarks

This case study of one KLS patient, for whom results were possible to obtain during both hypersomnic and asymptomatic periods, suggests that KLS might be associated with disrupted thalamo-pontine functional connectivity. The results also indicate that neuropathology in the dorsal pons might be a common factor behind both the patient’s nystagmus and her hypersomnia.

## Conflict of Interest Statement

The authors declare that the research was conducted in the absence of any commercial or financial relationships that could be construed as a potential conflict of interest.
